# The Gap in the Current Research on the Link between Health Locus of Control and Multiple Sclerosis: Lessons and Insights from a Systematic Review

**DOI:** 10.1155/2013/972471

**Published:** 2013-02-14

**Authors:** Nicola Luigi Bragazzi

**Affiliations:** School of Public Health, Department of Health Sciences (DISSAL), University of Genoa, Via Pastore 1, 16132 Genoa, Italy

## Abstract

Multiple sclerosis (MS) is a chronic neurological disease whose etiology has not been fully understood yet in detail. Empirical findings show how psychosocial symptoms are very important features of the clinical presentation of MS, having a deep impact on patient's quality of life, and thus psychological coping strategies may play a central role in reducing the burden of the disease and improving patient's satisfaction of life. MS progression and relapses/exacerbations are unpredictable and may depend on factors such as stressor chronicity, frequency, severity, type, and individual patient characteristics such as depression, personality, locus of control (LOC), optimism, and perceived social support. Due to its importance for health-care delivery, rehabilitation, and nursing, here, we make a systematic review on the current state-of-the-art studies concerning the relationship between LOC and MS, according to the PRISMA guidelines, and we assess the quality and the completeness of the studies using the CONSORT instrument, underpinning their limitations, and suggesting how to fill the gap in this research field.

## 1. Introduction

### 1.1. Multiple Sclerosis

Multiple sclerosis (MS) is a chronic autoimmune inflammatory progressive neurological disorder resulting in injury of the oligodendrocytes and in axonal demyelination [1]. There are at least 2–2.5 million patients worldwide suffering from MS, and its prevalence is unevenly distributed and highly variable from less than 5 cases per 100,000 inhabitants up to more than 100–200 cases per 100,000 inhabitants [2]. It is more likely to affect women than men with a ratio of 2.3 which has gradually increased over time, and the age of onset is generally young adulthood, usually affecting people in their 20s or 30s [3], even though pediatric and late onset as well as clinical variants have been reported.

The progress of the disease is extremely variable and unpredictable, the etiology is unclear, there is currently no cure, and only symptomatic therapy is available [1]. 

### 1.2. The Psychosocial Burden

Psychosocial symptoms are very important clinical features of the presentation of MS, having a deep impact on patient's quality of life [4], thus suggesting an integrated biopsychosocial approach [5, 6] to the disease, as also strongly recommended by the American MS task force [7]. Psychological symptoms include above all depression [8], with a rather high prevalence around 50%, as reported by the recent large-scale study of anxiety and depression prevalence in people with MS in the UK [9]. Depression is more common during relapses and seems to exacerbate other symptoms such as fatigue and cognitive dysfunction [9]. Anxiety, as well as perceived stress and psychological distress, is also frequent while other psychiatric illnesses occur less frequently in MS [10–13].

MS, being a chronic disease, has a deep impact on patient's life since it can impair patient's life goals, such as employment, income, and living daily activities as well as social relationships and interactions. Collected evidence indicates that the relationship between life stress and relapse is complex and is likely to depend on factors, such as stressor chronicity, frequency, severity, and type, and individual patient characteristics, such as depression, health locus of control (LOC), optimism [14], self-efficacy [15], spirituality [16], perceived social support [17], and coping strategy use [18]. Moreover, a quarter of MS patients suffer from suicidal ideation [19, 20], being the risk of suicide 5–10 higher than in the general population [19].

For these reasons, psychological symptoms in MS patients should not be underestimated and should be properly addressed by the clinicians, since an earlier diagnosis of these disorders could ameliorate patient's quality of life and MS course. Thus, studying the psychosocial burden of MS has a great pragmatical value and can help in designing an *ad hoc* psychosocial intervention.

Due to its importance for health-care delivery, rehabilitation, and nursing, here, we make a systematic review on the current state of art concerning the relationship between LOC and MS.

### 1.3. Health Locus of Control

LOC is the patient's belief in the location of the control over results of his/her behavior [20–23], that is to say a method or a strategy that patients use in order to attribute the cause of their own disease. An individual who thinks that he/she can determine events in his/her environment by his/her own actions is said to have an internally oriented LOC. The contrary orientation is referred as externality of LOC [24]. Internal LOC in fact correlates with volitional perception, an idea of a controllable (and curable) disease, while external LOC reflects the patient's impression of an incurable illness and a denial of its symptoms, which often leads to refusing care and rehabilitation [24].

LOC could be linked to stress, whose correlation with MS has been extensively investigated, in particular from a psychoneuroimmunological perspective. Stressful events can lead to an activation of endocrine (hypothalamus-pituitary-adrenal axis) and autonomic pathways, as well as to a dysregulation of immune system, thus influencing the relapse and the course of MS [25]. Even though a psychobiological explanation of LOC is lacking, there are some empirical studies that have shown a correlation between salivary immunoglobulin A (IgA) and LOC orientation [26], while other researchers have found internal LOC working as a “buffer” against the decrease of cellular immunity in depression (the so-called “immune control theory”) [27].

LOC has a profound impact on patient's behaviors, such as increased motivation in seeking health-related information, compliance, and adherence to the treatment, that are of fundamental importance in order to properly manage MS course. Thus, the understanding of LOC would help physicians to better treat MS patients, since psychological parameters could be modified through a psychotherapeutic intervention, while physical symptoms are more persistent and refractory to being adjusted. 

## 2. Materials and Methods

This systematic review was carried out according to the standards of Preferred Reporting Items for Systematic reviews and Meta-Analyses (PRISMA) guidelines [28], and the study protocol was registered in PROSPERO database (registration number: CRD42013003586, date of approved registration: January 8th 2013). MEDLINE/PubMed, PsycINFO, and ISI Web of Knowledge archives and databases were consulted and scanned, searching for a combination of keywords, namely “multiple sclerosis” and “health locus of control,” using Medical Subject Headings (MeSH) terms as vocabulary, according to the NCBI nomenclature and guidelines.

Inclusion criteria were (1) articles fully peer-reviewed, (2) articles with relevant quantitative details and information about the type of study (randomized clinical trial, matched case-control, cohort study, and so on), (3) clearly stated collected evidence and results, and (4) articles being written in English language.

Exclusion criteria were (1) conference proceedings and other not fully peer-reviewed material, (2) items not directly pertinent to the query string, (3) articles not containing sufficient information, and (4) articles not written in English language, which consequently were discarded.

The collected articles were rigorously assessed using the Consolidated Standards of Reporting Trials (CONSORT) instrument, developed by Turner et al. [29] and summarized in Table 1.

## 3. Results

Our initial query resulted in 47 hits (specifically, 18 articles from MEDLINE/PubMed, 2 from PsycINFO, and 27 from ISI Web of Knowledge), and after discarding the duplicated items the resulting list included 28 nonredundant articles. Only 9 studies were finally considered in our systematic review (3 articles were discarded being a review, 1 not being a fully peer-reviewed manuscript but a conference proceeding, 8 not being directly pertinent to MS, 4 not being written in English language, 1 not being sufficiently quantitative, 1 not being directly pertinent to the relationship between LOC and MS, but focusing on therapy, and 1 for sampling issues). The final list of selected articles is summarized in Table 2.

## 4. Discussion

Studying a randomly selected sample of 157 subjects, Garfield and Lincoln [30] interestingly found a rather high prevalence of anxiety among MS patients (about 56.7%), which correlated with gender, self-efficacy, depression, self-perceived stress, and disability. Years since diagnosis, months since the last relapse, and age were instead not statistically significant in correlation with anxiety. LOC was not found to be a significantly correlated variable.

Using the Weiner's causal attribution and control cognitions perceptual framework, Wells et al. [31] failed to find a connection between LOC and fatigue/pain perception. In a sample of 140 participants, fatigue threshold was correlated with causal attributions, control cognitions, and exercise frequency, but not with LOC.

Gay et al. [32] used the causal pathway analysis (developed by Sewell Right) to model the data obtained from assessing a sample of 115 patients and found that LOC could not be used to predict patient's psychological attitudes.

Vuger-Kovačić et al. [33] found that most MS patients exhibited external LOC and showed a correlation between LOC orientation and time since MS diagnosis as well as depression.

Using Smith's quality of life conceptual framework based on four components (namely, clinical health, role performance, adaptability, and wellbeing), Schwartz [34] investigated a pool of 136 randomly selected MS patients and repeated the measurements after 2, 12, 18, and 24 months. Schwartz compared two different kinds of psychological interventions: one being a coping skills teaching group, the other being a peer telephone support intervention, as offered by the National MS Society. The two groups differed in many aspects, such as in the degree of professionalism, commitment, and efforts, in the involvement of lay resources and people, and in how directive they were. The author found that the peer support intervention increased the externality of LOC but did not influence psychosocial role performance or wellbeing. 

L. Macleod and G. Macleod [35] investigated a very small MS sample (20 subjects) and did not find any correlation between internality of LOC and clinical and psychological variables.

Wassem [36] studied 100 randomly selected MS patients from a MS support group and found that LOC was a good predictor of patient's strategies, coping, level of knowledge, and level of self-care practices. Interestingly, LOC was not statistically correlated with time since the diagnosis.

Investigating a population of 60 MS patients, Halligan and Reznikoff [37] found that internal LOC was negatively correlated with depression and positively with body image and perception but was uncorrelated with disease duration or disability.

Brooks and Matson [38] investigated 103 MS patients and found that females were more likely to show positive adjustment. Subjects with an internal LOC had more positive adjustment scores.

## 5. Conclusion 

Despite the increasing body of research devoted to the psychological aspects of MS, the relationship between the disease and LOC has been poorly explored. 

Our research resulted in only 9 fully peer-reviewed studies, which however presented a different range of quality and academic rigor and some limitations. Only 1 study explicitly used a rigorous method (namely, the Dillman's total design method), including sample size effect calculation and properly estimating the minimum number of participants required to detect a significant difference among compared groups. Only 1 study reported the highest score possible using Moher's CONSORT assessment instrument. In the other studies the quality was rather poor. Most studies used self-reported questionnaires, without an external validation of a psychologist or a psychiatrist. This could also lead to a bias, excluding patients not able to compile questionnaires, such as those suffering from cognitive impairment. Thus, the generalization of these studies to larger samples and populations is questionable. Moreover, only in few studies the self-report scales concerning MS disabilities were confirmed by a neurologist or supported by clinical findings and neuroimaging.

Apart from the researches carried out by Schwartz [34] and by Brooks and Matson [38], which represent a longitudinal trial, all the other researches are cross-sectional, and studies of this kind are of difficult interpretation because it is hard to understand whether anxiety, depression, or other psychological strategies are a consequence of the disease (causal inference) or rather a reaction to the illness. 

Few studies are theory-driven and use a well-established conceptual framework, such as those by Wells et al. [31] and by Schwartz [34].

Other concerns are quite technical and are the following: the limited dimension of the samples (with the exception of the study by Vuger-Kovačić et al. [33], which included more than 400 patients, the other study sample size was less than 200, in the range 20–157), the possibility of incurring in type 1 error due to multiple analysis, the psychometric parameters of the used scales (such as in some cases the low consistency rate and the Cronbach's alpha coefficient). The control group was well established only in few studies, such as those by Brooks and Matson [38], which used an external control group of healthy subjects, with similar age and gender distribution.

On the basis of the evidences we collected we can conclude as follows.

1. Externality of LOC is a predictor of higher disabilities, depression, anxiety, and stress level; only three studies seem to contradict this conclusion, namely, the study by Garfield and Lincoln [30], the one by Gay et al. [32], and the study by L. Macleod and G. Macleod [35].
2. There seems to be a link between externality of LOC and self-reported symptoms as fatigue, as noted by Wells et al., 2012 [31].
3. Internality of LOC and good and positive representations of the disease are predictors of the patient's behaviors and attitudes, such as compliance and adherence to therapy and to social support request/access. Moreover internality of LOC is generally present in subjects with higher knowledge and self-awareness of their disease, who result to be more informed, practicing more self-care than those with an external LOC.
4. An interesting study by Brooks and Matson [38] suggests that LOC may be linked to gender, since they found that females were more likely to have an internal LOC than males.
5. There are very few studies focusing on the changes of LOC during the progression of MS; an important exception is the beautiful study carried out by Vuger-Kovačić et al. [33], who demonstrated a shift of LOC, which has not been found instead by Halligan and Reznikoff et al. [37] and by Wassem [36].
6. Another little studied topic is the relationship between LOC and the type of psychological support adopted by the physicians. Practitioners should be aware that the methodology of psychological help can have an important influence on the externality of LOC, as shown by Schwartz [34]. In this study teaching how to enhance adaptive attitudes and coping skills (addressing emotional impairment, difficulties in mood, cognitive deficits, and communications with the caregivers) resulted in a better health outcome, while less favorable results were obtained with the help of peer telephone support groups based on Rogerian client-centered psychotherapy. The former intervention exploited trained professionals, required a high degree of commitment and efforts, being highly directive and bilateral, and the latter made use of lay people and resources and was not directive. However from a subgroup analysis, it emerged that the peer support group was more helpful for MS patients suffering from depression or other affective problems than the other psychological intervention. Other studies have shown that supportive psychotherapy, modulating and enhancing patient's self-efficacy, can result in a better health outcome and self-reported quality of life, as shown by Riazi et al. [39]. Combined pharmacological treatment and psychological/social/occupational support are referred to as multidisciplinary intervention (MI). Research in other areas has offered evidence that MI can change and modulate health-related outcomes, including LOC, for example, in the treatment of fibromyalgia [40] or chronic stage of stroke [41], but the effects of MI on MS patients have been poorly investigated, with a lack of evidence [42].

Further research will be needed for a better understanding of the role played by LOC in MS patients and for providing a better healthcare service. Future studies should be carried out according to a standardized randomized protocol, recruiting a large sample, defining clearly inclusion/exclusion criteria, establishing control groups, and using a solid conceptual framework [42].

## Figures and Tables

**Table 1:** Grid used for assessing the papers (adapted from Moher).

Category	Description	Grading
A	Sample size calculation, estimating the minimum number of participants required to detect a significant difference among compared groups	0 = did not exist/not mentioned/not clear 1 = was reported but not confirmed 2 = reported and confirmed

B	Randomization and allocation concealment methods	0 = clearly inadequate 1 = possibly adequate 2 = clearly adequate

C	Clear definition of inclusion and exclusion criteria	0 = no 1 = yes

D	Completeness of follow-up (specified reasons for withdrawal and drop-outs for each group)	0 = no/not mentioned/not clear 1 = yes/no withdrawals or drop-outs occurred

E	Experimental and control groups comparable at study baseline for important prognostic factors	0 = no 1 = unclear/possibly not comparable for one or more important prognostic factors 2 = clearly adequate

F	Presence of masking	0 = no 1 = unclear/not complete 2 = yes

G	Appropriate statistical analysis	0 = no 1 = unclear/possibly not the best method applied 2 = yes

**Table 2:** All articles collected for the systematic review.

Authors	Methods	Results	Quality assessment
Garfield and Lincoln, 2012 [30]	Cohort study, with 157 participants who decided to take part in the study out of 400 randomly selected from a database of 1144 patients, aged 32–90 years, and 30% men and 70% women. Patients with EDSS ≥6.5 were excluded. They were asked to complete self-report questionnaires concerning self-efficacy (MSSS) and LOC (MHLC), depression (HADS), anxiety, general stress and psychological distress (PSS), and disability (GNDS). Moreover, they were asked to provide clinical information specifically relevant to their current disease status. Control group was given by non-anxious MS patients.	Anxiety was the primary health outcome. 89 (56.7 %) subjects were clinically anxious, showing the following: (1) higher level of disability (*P* value < 0.001); (2) lower level of self-efficacy (*P* value < 0.001); (3) higher level of depression (*P* value < 0.001); (4) higher level of stress (*P* value < 0.001). LOC was *not* a predictive variable of clinical anxiety.	A = 0 B = 2 C = 1 D = 1 E = 2 F = 2 G = 2

Wells et al., 2012 [31]	Cohort study with 140 participants (97 females and 43 males, aged 18–83 years). They were asked to compile a self-reported questionnaire concerning control cognitions and causal attribution (RCDS-II, CAL), LOC (MHRLOC), perceived fatigue (FSS), and coping (WOCQ). Moreover, they were asked about exercise frequency.	LOC had a mixed influence on fatigue threshold and perception. When the causes of fatigue were perceived as external, and stable, uncontrollable, participants reported higher fatigue scores. However, this was not statistically significant and moreover the scales RCDS-II and CAL gave contradictory results. Fatigue threshold instead correlated with psychosocial cognitions and attributions as well as with lifestyle, exercise frequency, and coping.	A = 0 B = 2 C = 1 D = 1 E = 2 F = 2 G = 2

Gay et al., 2010 [32]	Cohort study with 115 participants (36 men and 79 women, aged 27–80 years). They were asked about their sociodemographic, medical, and psychological characteristics by completing dedicated questionnaires about disabilities (EDSS), depression (Zung rating score), anxiety (STAI), coping (CHIP), social support (SSQ6), LOC, alexithymia (TAS-20), and self-esteem (SEI).	25.9% of the participants reported high depression scores, while 36.3% of the subjects were anxious. Functional status (EDSS), trait anxiety, alexithymia, and satisfaction with social support system were predictive factors of depression. LOC was *not* a direct predictive factor.	A = 0 B = 0 C = 0 D = 0 E = 1 F = 0 G = 2

Vuger-Kovačičet al., 2007 [33]	Cohort study with 457 participants. They were asked to answer to the locus of control inventory (Croatian version of Rotter's scale) and CCEI questionnaire of personality. The sample was subdivided into 3 groups, according to time since the diagnosis. No clear information about age and gender is given in the article.	405 (88.6%) MS patients exhibited external LOC. As the disease progressed, LOC shifted from internality to externality. Depression and anxiety sub-scales increased too, in a statistically significant way. Statistical analysis confirmed the hypothesized relationship between external LOC and anxiety, depression and maladaptive behavior.	A = 0 B = 0 C = 0 D = 0 E = 1 F = 0 G = 1

Schwartz, 1999 [34]	2-year longitudinal trial with 132 MS patients, randomly selected from an initial list of 172 subjects, with a mean age of 43 years ± 9, 73% women, and 27% men, comparing a coping skills teaching group (*n* = 64) with a peer telephone support group (*n* = 68). MS patients with EDSS ranging from 1 to 8.5 were included. Psychotic patients or those with cognitive impairment were excluded. Subjects were asked to fill in different questionnaires about perceived fatigue (SIP, MAFS), self-reported health status, LOC (MHLC), coping strategies (WCC), self-efficacy (MSSE), and quality of life and wellbeing (AIMS). They were also assessed with neuropsychological tests (the Rao cognitive battery, the Wisconsin Card Sorting Task, and the Trail Making Test).	The peer support intervention increased the externality of LOC and the use of blameful coping strategies but did not influence psychosocial role performance or wellbeing. Instead, the coping skills teaching group increased the internality of LOC and the use of reframing coping strategies, as well as social activity, satisfaction with family, and global satisfaction scores.	A = 2 B = 2 C = 1 D = 1 E = 2 F = 2 G = 2

L. Macleod and G. Macleod, 1998 [35]	Matched case-control study with 25 subjects aged 29–58 years, 36% men and 64% women. LOC beliefs were investigated in terms of their relationship with anxiety and depression, using the RLOC, the BDI, the STAI, the pain self-perception scale (WHYMPI), and the Barthel ADL. The matched comparison group was given by spinal cord injury patients (SCI). Barthel ADL was used to create subgroups.	SCI patients were more internally oriented than MS subjects. However, internality of LOC was *not* linked to lower levels of depression or anxiety.	A = 0 B = 0 C = 0 D = 1 E = 1 F = 0 G = 2

Wassem, 1991 [36]	Randomized study, with 100 participants (aged 21–78 years) randomly selected from the membership list of a state MS support group. No information is available about the gender distribution. The participants were asked to compile self-report questionnaire about disabilities (the Kurtzke DSS) and LOC (HLOC). A further questionnaire was developed by the author, concerning the level of knowledge and self-care practices. The sample was subdivided in two categories: referred as “internal” (exhibiting internality of LOC) and “external” (exhibiting externality of LOC) patients.	Subjects with an internal LOC were more aware and informed about their disease, performed more self-care, and had a more benign course of MS. Time since the diagnosis was not statistically significant.	A = 0 B = 2 C = 0 D = 0 E = 2 F = 2 G = 2

Halligan and Reznikoff 1985 [37]	Cross-sectional study with 60 22–72-year-old patients, 18 men and 42 women. They were asked about their body image (the Holtzman inkblots) and representation, depression (PERI), and locus of control (using the Rotter's Internal-External LOC Scale). Moreover, sociodemographic parameters (sex and age) and clinically relevant information (duration of disease and degree of disability) were investigated.	Internal LOC was negatively correlated with depression and positively with body image and perception, but was uncorrelated with disease duration or disability.	A = 1 B = 2 C = 0 D = 1 E = 2 F = 1 G = 2

Brooks and Maston, 1982 [38]	Longitudinal study with 103 participants (mean age 52 years, 68% women). They were asked about sociodemographic, disease-related, medical parameters (physical mobility, degree of impairment), and social-psychological variables (self-concept instrument). The authors elaborated *ad hoc* indexes (like EMSR, LOCONTR, MOBDIF, SCALDIF, SCALT, and SYMPINDX). Control group is given by a sample of healthy subjects, with similar age, gender distribution.	Females were more likely to show positive adjustment. Subjects with an internal LOC had more positive adjustment scores.	A = 0 B = 2 C = 1 D = 1 E = 2 F = 2 G = 2

ADL: activities of daily living; AIMS: Arthritis Impact Measurement Scale; BDI: Beck Depression Inventory; CAL: Causal Attribution List; CCEI: Crown-Crisp Experiential Index; CHIP: coping about health injuries and problems; DSS: Disabilities Status Scale; EDSS: Expanded Disability Status Scale; EMSR, effect of MS on social relationships; FSS: Fatigue Severity Scale; GHQ-12: General Health Questionnaire; GNDS: Guys Neurological Disability Scale; HADS: Hospital Anxiety and Depression Scale; HLOC: health locus of control; LOCONTR: locus of control; MAFS: Multidimensional Assessment of Fatigue Scale; MHLC: multidimensional health locus of control; MHRLOC: multidimensional health-related locus of control; MOBDIF: change in mobility; MSSE: multiple sclerosis self-efficacy; MSSS: Multiple Sclerosis Self-efficacy Scale; PERI: psychiatry epidemiology research interview; PSS: Perceived Stress Scale; RCDS-II: Revised Causal Dimension Scale; RHS: Ryff Happiness Scale; RLOC: recovery of locus of control; SCALDIF: self-concept over time; SCALT: self-concept; SYMPINDX: Symptom Index; SEI: Self-Esteem Inventory; SIP: Sickness Impact Profile; SSQ6: Social Support Questionnaire; STAI: Spielberger Trait Anxiety Inventory; TAS-20: Toronto Alexithymia Scale; WCCC: Way of Coping Checklist; WHYMPI: West Haven-Yale Multidimensional Pain Inventory; WOCQ: Ways of Coping Questionnaire.
